# Help-seeking behaviors among Chinese people with mental disorders: a cross-sectional study

**DOI:** 10.1186/s12888-019-2316-z

**Published:** 2019-11-29

**Authors:** Huifang Yin, Klaas J. Wardenaar, Guangming Xu, Hongjun Tian, Robert A. Schoevers

**Affiliations:** 1 Tianjin Mental Health Institute, Tianjin Anding Hospital, No. 13, Liulin Road, Hexi district, Tianjin, 300222 China; 2 Department of Psychiatry, Interdisciplinary Center Psychopathology and Emotion regulation (ICPE), University of Groningen, University Medical Center Groningen, Hanzeplein 1, 9713 GZ Groningen, The Netherlands

**Keywords:** Help seeking, China, Mental healthcare, Mental disorder, Treatment

## Abstract

**Background:**

Failure to seek treatment for mental health disorders is a serious public health concern. Unfortunately, there is little insight into help-seeking and its associated factors in China which has undergone rapid economic development in the past 30 years and has an increasing prevalence of mental disorder. Therefore, this study aimed to (1) investigate help-seeking rates in healthcare and non-healthcare settings and (2) investigate the correlates of help-seeking behavior in a large Chinese survey.

**Methods:**

Data came from the Tianjin Mental Health Survey (TJMHS), a representative sample of adult community residents in the Chinese municipality of Tianjin (*n* = 11,748). Of these, 1759 individuals had ≥1 axis-I diagnosis according to the Diagnostic and Statistical manual– fourth edition (DSM-IV) and were administered a Help-Seeking Questionnaire.

**Results:**

15.7% of patients reported that they had ever sought help during their entire lifetime before the interview, with 4.5% seeking help in mental healthcare, 3.2% in other healthcare and 8.1% in non-healthcare settings (e.g., family, friends, and spiritual advisor). Among help-seekers, the first help was mostly sought in non-healthcare settings (58.4%), followed by healthcare (27.5%) and mental healthcare settings (24.5%). Female gender, younger age, having 7–9 years vs 0–6 years of education, a low income, a psychotic disorder and having ≥2 disorders were associated with increased help-seeking. Older age, being married and having a psychotic or organic disorder were associated with increased help-seeking in healthcare vs. non-healthcare settings.

**Conclusion:**

A small percentage of persons with mental disorders in the Tianjin region seek help and among those who do, variations in the types of help-seeking may be partially explained by demographic and clinical characteristics.

## Background

Although mental disorders contribute significantly to global health problems and cause a severe burden on both patients and their environment, many individuals who have or experience a mental disorder do not seek treatment, especially in low- and middle-income countries, including China [1, 2]. Previous surveys in China have shown that the treatment-seeking rates of individuals with a mental disorder are low (12-month rate: 3.4%) [3] and lower than rates observed in other low and low-middle income countries [4]. It has been suggested that these comparatively low rates of mental healthcare use in China might be due to inadequate resources to meet demands, unequal distribution of mental health services across urban and rural areas, and/or inadequate training of the mental health workforce [5].

To address the problem of low mental healthcare use, Chinese healthcare policy is set to focus on increasing the availability of trained healthcare and non-healthcare workers to improve the effective delivery of mental healthcare. However, to gain better insight into the need for mental healthcare and optimal targeting of improvements, the patterns of help-seeking behaviors of mental health patients and the correlates of these patterns should be investigated more closely [6]. Surveys of help-seeking behavior in Shenzhen and in Beijing and Shanghai showed help-seeking rates of, respectively, 6.7 and 2.9% from healthcare services and of, respectively, 4.6 and 1.0% from non-healthcare services [3, 7]. Several studies have investigated correlates of help-seeking and have shown that the experience of high psychiatric stigma, low mental health knowledge, mild severity of mental disorders, being separated, divorced or widowed, having a low-income status, and living in a rural area are associated with a lower probability of seeking and receiving any help for mental disorders in China [3, 8, 9].

Although previous work has provided important insights into the general patterns and correlates of help-seeking in China, several important points are in need of closer investigation. First, little is known about the distribution of help seeking across different kinds of sources and/or providers of care. Second, the roles of lay workers or non-formal care providers have been ignored in previous Chinese studies, although it is known that these play an important role in helping people with mental health problems [5, 10, 11]. Third, previous studies on associated factors of help-seeking have mainly looked at demographic factors, whereas clinical factors, such as a patient’s diagnosis and the disorder’s severity may also be associated with help-seeking behavior [12]. Finally, the relationships between mental illness stigma and mental health knowledge (MHK), on the one hand, and help-seeking behavior, on the other hand, have so far received little attention in China, whereas the former are both considered barriers to help seeking for individuals with mental problems [5, 8, 10].

Addressing above mentioned points will contribute to gaining the specific insights that are required to guide the development of a better community-based mental healthcare system that integrates hospital and community mental services into the general healthcare system in China [6, 13]. Therefore, this study aimed to investigate: (1) the total help-seeking and first-time help-seeking rates from different types of help/healthcare providers among individuals with mental health disorders living in community, (2) the clinical (e.g., severity; diagnosis) and demographic factors related to help-seeking and different types of help/healthcare, and (3) the associations of help-seeking behavior with perceived stigma and MHK.

## Methods

### Sample and procedures

Data came from the Tianjin Mental Health Survey (TJMHS; *n* = 11,748) conducted between July 2011 and March 2012 in Tianjin. A detailed description of the survey design can be found elsewhere [14]. In short, the TJMHS used a two-phase design and a multistage cluster random sampling method to select a large, representative community sample of respondents aged 18 years and older in Tianjin. In the first phase, 11,748 subjects were screened using an expanded version of the 12-item General Health Questionnaire (GHQ-12) for psychopathology risk. Second, Based on screening with the expanded GHQ, 56.6% of respondents were classified as low-risk of a mental disorder, 20.4% as moderate-risk, and 23.0% as high-risk. All high-risk individuals, a 45.7% random sample of moderate-risk individuals, and an 11.5% random sample of low-risk individuals were selected for participation in the Phase 2 diagnostic assessment. In the second phase, 4438 participants of the 4563 selected individuals participated in the interview with the Structured Clinical Interview for the Diagnostic and Statistical Manual – fourth edition (DSM-IV) axis I disorders (SCID) and also a help-seeking questionnaire (see below) even when they did not meet a DSM diagnosis according to the SCID. Of the 4438 interviewed participants, 1759 individuals met criteria for one or more DSM-IV axis I mental disorder diagnoses according to SCID [15] and were included in the current study to evaluate their help-seeking behavior and the associated demographic and clinical factors.

One of the aims of the TJMHS is to assess the mental health knowledge and mental health stigma among community members. Because it was time consuming for all participants to complete all questionnaires, we administered the additional questionnaires only in a selected subsample. According to sample size calculations, about 12% of participants in the phase 1 screening should be selected for this to ensure a sufficient sample size. In the currently used sample of patients (*n* = 1759) these measures were available for 238 patients (13.5%).

Each SCID was conducted by a psychiatrist interviewer, who completed the screening and diagnostic assessment. In total, 44 psychiatrists participated in the TJMHS SCID interviews. They all had two training sessions: a 5-day initial training session about the design of the project and the screening procedures and a rigorous 15-day training session in the administration of the household structure form and the screening and diagnostic instruments. Scales were read by a psychiatrist interviewer to the subject because reading might be a problem for some participants to complete these scales by themselves. If a participant did not understand the meaning of the words in the standard questions, psychiatrists were allowed to explain the meaning of the standard questions to help the participant.

### Measures

Most of the used measurement instruments are originally self-report questionnaires. However, in the TJMHS all questionnaires were interviewer-administered because a considerable part of the sample was expected to be illiterate or semi-literate. To make sure that measurement was standardized across respondents, the same assessment method was also used in all literate subjects.

#### The 12-item general health questionnaire

The Chinese GHQ-12 [16] is used to assess general psychological distress in the past 30 days. Respondents with a GHQ-12 score above 3 were considered to have some mental problems. The Chinese GHQ-12 has adequate internal consistency (alpha = 0.75) and test-retest reliability (0.72) [16].

#### Structured clinical interview for the diagnostic and statistical manual

The Chinese version of the SCID [17] was administered by certified psychiatrists to assess the presence of DSM-IV axis-I mental disorder diagnoses. Most of the diagnoses can be recorded as ‘lifetime’ (i.e. meeting diagnostic criteria during the participants’ entire lifetime before the interview) or ‘1-month’ (i.e., meeting diagnostic criteria at any time during the month before the interview). The Chinese SCID has previously been shown to be reliable and valid [8]. In the current study, diagnoses were pooled into six broader categories: (i) mood disorders, (ii) anxiety disorders, (iii) substance use disorders, (iv) psychotic disorders, (v) organic mental disorders and (vi) other mental disorders. The specific disorders included in each of these categories are shown in Additional file 1. In the TJMHS, the test-retest reliability of the SCID was tested (see [14] for details) and kappa values for a 1-month diagnosis (yes/no) were 0.93 for psychotic disorders, 0.64 for organic mental disorders, 0.76 for affective disorders, 0.82 for anxiety disorders, and 0.80 for substance abuse disorders.

#### Help-seeking questionnaire

A detailed questionnaire which was developed by Michael Phillips to assess respondents’ actual help-seeking behavior for psychological problems was included in the expanded Chinese version of the SCID [17]. This instrument lists 23 possible sources of help for psychological problems. For each source of help, participants were asked if they had ever used it for problems with emotions, nerves, mental health, the use of alcohol or drugs or other mental health related problems (response scale: ‘yes’/‘no’). In this study, the different sources of help were divided into two categories: *healthcare* services and *non-healthcare* services. Healthcare services included mental healthcare services and non-mental healthcare services. Assessed mental healthcare services were: a psychiatric clinic in a general hospital, a regular clinic in a psychiatric hospital, a specialized clinic in a psychiatric hospital, inpatient treatment in a psychiatric hospital, and a community psychotherapy institute. Assessed non-mental healthcare services were: a private doctor of western medicine, a private doctor of Traditional Chinese Medicine (TCM), an internal medicine clinic in a general hospital, a neurology clinic in a general hospital, inpatient treatment in a general hospital, an outpatient clinic in a TCM hospital, inpatient treatment in a TCM hospital, a community health center, and a community pharmacy. The assessed non-healthcare services/sources were: relatives, colleagues/friends/neighbors, a witch doctor, a Qigong practitioner, a temple, writing letters to get counselling, a newspaper article or magazine, an internet support group, and a hotline. If a subject reported seeking more than one source of help, it was asked which help they sought first. The TJMHS was the first study in which this questionnaire is implemented after a pilot study in two communities (*n* = 1000) [14].

#### The perceived discrimination and devaluation scale and mental health knowledge questionnaire

The Perceived Discrimination and Devaluation scale (PDD) [18] is a 12-item questionnaire assessing a respondent’s expectations of devaluation and discrimination toward current or former psychiatric patients. The items ask respondents how they think ‘most people’ or ‘most employers’ think or act toward persons with a current or a prior psychiatric disorder. The current study used the Chinese version of the PDD, which has the same items as the original but uses a slightly different response scale, adding the option ‘not sure’. This version of the PDD was previously shown to have acceptable psychometric properties [19].

The MHK Questionnaire (MHKQ) was developed by the Ministry of Health of China and was used to assess MHK. The scale consists of sixteen items with a dichotomous response scale (‘yes’/‘no’). A higher total score (range: 0–16) indicates higher MHK. The items of the MHKQ are shown in Additional file 2 [14].

#### Global assessment of functioning

The Global Assessment of Functioning (GAF) [20] was conducted by the same psychiatrist who administered the SCID to rate the level of dysfunction in the previous month due to mental illness. A disability weight was estimated using the GAF score (disability weight = [100-GAF score]/100), and individuals with a disability weight of 0.40 or greater were defined as ‘moderately to severely disabled’.

### Statistical analyses

Participants were first divided into a help-seekers group and non-help seekers group. In the help-seekers group, a division was then made between healthcare help-seekers and non-healthcare help-seekers. Cross-tabulation was conducted to investigate the percentages of help-seekers and non-help-seekers and to compare healthcare and non-healthcare help-seekers. To investigate which factors were associated with help-seeking among persons with a mental disorder diagnosis, a series of univariable logistic regression analyses were conducted, each using the dichotomous help-seeking outcome (no help-seeking vs. help-seeking) as dependent variable and one of the demographic factors and diagnostic categories as independent variable. The variables with significant associations in these univariable logistic regression analyses were included in a multivariable logistic regression model to estimate their independent effects and gain insight into their combined effect on help seeking behavior. Next, similar analyses were carried out in the subsample of help-seekers, using the type of help (healthcare vs. non-healthcare) as outcome. Finally, to investigate the association of stigma and MHK with help-seeking in the subsample of patients that completed the PDD and MHKQ, Mann-Whitney U Tests were used to compare PDD and MHL scores between help-seeking groups. A *p*-value < 0.05 was considered to indicate a statistically significant effect. IBM SPSS Statistics Version 25 was used to analyze the data. Weights were used in all statistical analyses (except the non-parametric analyses with the PDD and MHK because the small sample size after weighting). The detailed weighting process was described previously [14].

## Results

### Sample characteristics

There were 1795 individuals diagnosed with any mental disorder. The weighted mean age was 44.2 years (SD = 15.9) and 38.8% of the sample was female. All other sample characteristics are shown in Table 1.

**Table 1:** The characteristics of participants (*N* = 1759)

Subject characteristics		N (unweighted)	%^a^
*Demographic characteristics*			
Female Sex		933	38.8
Age groups	18–39	339	43.6
	40–54	560	29.0
	55+	860	27.4
Resident region	Urban	1288	80.3
	Rural	471	19.7
Marital status	Never married	130	18.7
	Married	1279	73.7
	Divorced/lost spouse	350	7.7
Years of education	0–6	564	19.4
	7–9	568	28.7
	10–12	368	24.4
	13+	259	27.5
Employment status	Housewife	145	6.4
	Employed	590	53.0
	Retired	555	16.8
	Unemployed/lost job	243	13.3
	Farmer	226	10.5
Income group	Below median or do not know	977	47.9
	Above median	782	52.1
Living status	Living alone	275	9.9
	Living with other people	1484	90.1
*Mental health characteristics*			
GHQ-12 score	< 4	1220	80.9
	≥4	539	19.1
GAF disability	Moderate to severe	442	16.9
	Mild	1317	83.1
Mood disorders		856	39.4
Anxiety disorders		359	19.2
Substance use disorders		459	37.1
Psychotic disorders		80	3.9
Organic mental disorders		206	7.7
Other mental disorders		56	2.7
Only NOS disorder		429	24.5
More than 1 diagnosis		282	11.4

^a^Weighted percentages

GHQ-12 = General Health Questionnaire; GAF = Global Assessment of Functioning; NOS=Not Otherwise Specified

### Prevalence of help seeking

The help-seeking rates among the individuals with a mental disorder diagnosis are shown in Table 2. Of the participants with mental disorders, 15.7% reported having ever sought any help during their entire lifetime before the interview. About 4.5% had sought mental healthcare services and 3.1% had sought other forms of healthcare. Approximately 8.1% of individuals had only sought help from non-healthcare care providers/services. The most common form of help sought was that of colleagues/friend/neighbors (7.2%) followed by relatives (5.5%). The top five most used healthcare sources were a regular clinic in a psychiatric hospital (2.9%), inpatient treatment in a psychiatric hospital (1.5%), an outpatient clinic in a TCM hospital (1.3%), an internal medicine clinic in a general hospital (1.2%) and a psychiatric clinic in a general hospital (1.1%). Of the individuals who had ever sought any help, the majority of individuals first sought help from non-healthcare sources (58.4%), with 54% seeking help from relatives and colleagues/friends/neighbors. About 27.5% first sought non-mental health services and 24.5% first sought mental healthcare. Of the sought healthcare services, a regular clinic in a psychiatric hospital was the most common first choice (13.5%), followed by an internal medicine clinic in a general hospital (5.5%), an outpatient patient clinic in a TCM hospital (5.5%), an inpatient treatment in psychiatric hospital (4.1%), and a psychiatric clinic in a general hospital (3.9%).

**Table 2:** The help-seeking rate (N = 1759) and first help- seeking rates (*N* = 323) for various sources

Help seeking sources		Any help (*n* = 1759)		First help (*n* = 323)	
		N (unweighted)	%	N (unweighted)	%
1.	Relatives	100	5.6	80	26.8
2.	Colleagues/friend/neighbors	101	7.2	59	27.4
3.	A private doctor of western medicine	15	0.8	11	3.3
4.	A private doctor of Traditional Chinese Medicine (TCM)	8	0.3	3	0.9
5.	A witch doctor	17	0.5	6	0.7
6.	A Qigong practitioner	2	0.1	0	0
7.	An internal medicine clinic in a general hospital	33	1.2	20	5.5
8.	A neurology clinic in general hospital	20	0.6	14	2.2
9.	A psychiatric clinic in a general hospital	31	1.1	16	3.9
10.	Inpatient treatment in a general hospital	6	0.2	2	0.4
11.	An outpatient clinic in a TCM hospital	37	1.3	23	5.5
12.	Inpatient treatment in a TCM hospital	0	0	0	0
13.	A regular clinic in a psychiatric hospital	72	2.9	49	13.5
14.	A specialized clinic in a psychiatric hospital	4	0.2	1	0.1
15.	Inpatient treatment in a psychiatric hospital	28	1.5	11	4.1
16.	A community psychotherapy institute	4	0.2	2	0.6
17.	A community health center	15	0.2	12	1.1
18.	A community pharmacy	4	0.1	4	0.6
19.	A temple	4	0.1	2	0.2
20.	Writing letters to get counselling	0	0.0	4	2.1
21.	A newspaper article or magazine	5	0.4	0	0
22.	An internet support group	2	0.0	1	0.1
23.	A hotline	0	0.0	0	0
	Other	7	0.4	3	1.1
Any form of help^a^		323	15.7	323	100
Any healthcare services^b^		201	7.6	168	41,6
	Mental healthcare services^c^	114	4.5	79	24.5
	Only non-mental healthcare services^d^	87	3.1	89	27.5
Only non-healthcare sources^e^		122	8.1	155	58.4

^a^Includes all forms of help listed in table

^b^Includes mental healthcare services and non-mental healthcare services

^c^Mental healthcare services include a psychiatric clinic in a general hospital, a regular clinic in a psychiatric hospital, a specialized clinic in a psychiatric hospital, inpatient treatment in a psychiatric hospital, a community psychotherapy institute

^d^Non-mental healthcare services include a private doctor of western medicine, a private doctor of Traditional Chinese Medicine (TCM), an internal medicine clinic in a general hospital, a neurology clinic in general hospital, inpatient treatment in a general hospital, an outpatient clinic in a TCM hospital, inpatient treatment in a TCM hospital, a community health center, a community pharmacy

^e^Non-healthcare sources include relatives and colleagues/friend/neighbors, a witch doctor, a Qigong practitioner, a temple, writing letters to get counselling, a newspaper article or magazine, an internet support group, and a hotline

### Demographic and diagnostic correlates of help seeking

Table 3 shows the associations of demographic and diagnostic characteristics with help-seeking. Being female, having a psychotic disorder and having ≥2 disorders were associated with higher odds of help-seeking. In the multivariable analysis, being in the older age-groups, having 7–9 years of education, and having an above median income were associated with lower odds of help-seeking compared to no help seeking. Table 4 shows the associations of demographic and diagnostic characteristics with help-seeking in healthcare settings compared to help-seeking in non-healthcare settings. In the multivariable analyses, being in the oldest age group, being married, having a psychotic disorder and having an organic disorder were significantly associated with higher help-seeking in healthcare services.

**Table 3:** Association between demographic and psychiatric characteristics and help-seeking (yes/no)

Characteristics		Univariable, OR (95% CI)	Multivariable, OR(95% CI)
Sex	Male	1	1
	Female	**3.23 (2.28–4.56)**	**2.89 (1.81–4.62)**
Age group	18–39	1	1
	40–54	**0.40 (0.26–0.61)**	**0.38 (0.22–0.66)**
	55+	**0.42 (0.27–0.65)**	**0.25 (0.12–0.53)**
Resident area	Urban	1	–
	Rural	1.13 (0.75–1.71)	–
Marital status	Never married	1	1
	Married	**0.38 (0.26–0.55)**	0.69 (0.41–1.17)
	Divorced/lost spouse	0.53 (0.27–1.03)	0.61 (0.26–1.45)
Years of education	0–6	1	1
	7–9	**0.56 (0.34–0.92)**	**0.47 (0.25–0.88)**
	10–12	1.03 (0.64–1.63)	0.95 (0.50–1.80)
	13+	0.67 (0.41–1.08)	0.61 (0.30–1.24)
Employment status	Housewife	1	1
	Employed	**0.51 (0.28–0.94)**	0.95 (0.46–1.96)
	Retired	**0.48 (0.24–0.98)**	1.60 (0.67–3.82)
	Unemployed/lost job	0.99 (0.50–1.96)	0.92 (0.42–2.03)
	Farmer	0.51 (0.23–1.11)	0.53 (0.22–1.31)
Per capita family income	Below median or do not know	1	1
	Above median	**0.46 (0.33–0.65)**	**0.58 (0.37–0.91)**
Living status	Living alone	1	–
	Living with other people	1.98 (0.99–3.97)	–
GHQ score	0–3	1	1
	4+	**2.04 (1.4–2.98)**	1.25 (0.80–1.96)
GAF disability	Mild	**1**	1
	Moderate to severe	**2.56 (1.74–3.75)**	1.43 (0.84–2.43)
Mood disorders	no	1	1
	yes	**1.79 (1.28–2.50)**	1.10 (0.71–1.73)
Anxiety disorders	no	1	–
	yes	1.32 (0.88–1.97)	–
Substance use disorders	no	1	1
	yes	**0.22 (0.14–0.35)**	0.54 (0.29–1.01)
Psychotic disorders	no	1	1
	yes	**11.44 (5.89–22.22)**	**9.44 (4.19–21.26)**
Organic mental disorders	no	1	–
	yes	1.40 (0.79–2.48)	–
Other mental disorders	no	1	–
	yes	1.93 (0.82–4.55)	–
Only NOS disorder	No	1	–
	yes	1.23 (0.84–1.78)	–
Number of diagnoses	1	1	1
	2 or more	**2.32 (1.49–3.61)**	**2.5 (1.40–4.45)**

Confidence intervals in bold type as statistically significant at the *p* < 0.05 level; GHQ-12 = General Health Questionnaire; GAF = Global Assessment of Functioning; NOS=Not Otherwise Specified

**Table 4:** Association between demographic and psychiatric characteristics and healthcare help-seeking

Characteristics		Univariable, OR (95% CI)	Multivariable, OR(95% CI)
Sex	Male	1	1
	Female	**0.46 (0.24–0.88)**	0.48 (0.17–1.42)
Age group	18–39	1	1
	40–54	**3.94 (1.68–9.26)**	3.19 (0.96–10.58)
	55+	**5.72 (2.3–14.26)**	**6.74 (1.58–28.68)**
Resident area	Urban	1	
	Rural	1.53 (0.72–3.25)	
Marital status	Never married	1	1
	Married	**2.65 (1.32–5.31)**	**8.63 (1.42–52.49)**
	Divorced/lost spouse	2.14 (0.64–7.20)	1.32 (0.12–14.8)
Years of education	0–6	1	1
	7–9	0.67 (0.25–1.82)	1.45 (0.35–6.06)
	10–12	**0.19 (0.08–0.48)**	0.86 (0.21–3.55)
	13+	**0.11 (0.04–0.30)**	1.35 (0.30–6.02)
Employment status	Housewife	1	
	Employed	0.56 (0.19–1.67)	
	Retired	3.27 (0.84–12.66)	
	Unemployed/lost job	1.26 (0.38–4.17)	
	Farmer	4.63 (0.93–23.01)	
Per capita family income	Below median or do not know	1	
	Above median	0.69 (0.36–1.31)	
Living status	Living alone	1	
	Living with other people	1.61 (0.41–6.26)	
GHQ-12 score	0–3	1	
	4+	0.82 (0.42–1.61)	
GAF disability	mild	1	1
	moderate to severe	**16.00 (6.22–41.21)**	2.96 (0.77–11.32)
Mood disorders	no	1	1
	yes	**0.43 (0.23–0.80)**	1.74 (0.62–4.87)
Anxiety disorders	no	1	
	yes	0.61 (0.29–1.29)	
Substance use disorders	no		
	yes	0.43 (0.17–1.12)	
Psychotic disorders	no	1	1
	yes	**118.75 (4.05–3483.86)**	**187.28 (4.19–8376.37)**
Organic mental disorders	no	1	1
	yes	**29.72 (2.56–345.42)**	**107.59 (4.39–2636.76)**
Other mental disorders	no	1	
	yes	0.46 (0.09–2.25)	
Only NOS disorder	No	1	1
	yes	**0.10 (0.04–0.25)**	2.40 (0.73–7.89)
Number of diagnoses	1	1	
	2 or more	1.76 (0.81–3.82)	

Confidence intervals in bold type as statistically significant at the *p* < 0.05 level; GHQ-12 = General Health Questionnaire; GAF = Global Assessment of Functioning; NOS=Not Otherwise Specified

### Help seeking, perceived stigma and mental health knowledge

Median scores of PDD in non-help-seekers (*n* = 196) and help-seekers (*n* = 42) were 37 (Interquartile range [IQR]: 37–43) and 38 (IQR: 34–44), indicating that there was no significant difference in terms of mental illness stigma. Median MHKQ scores showed a small, but significant difference between non help-seekers and help-seekers (11 [IQR: 10–12] vs. 12 [IQR: 10–14], *p* = 0.025). There were no statistically significant differences in mental illness stigma or MHKQ scores between help seekers in healthcare and non-healthcare settings.

## Discussion

The results of the current survey showed that of individuals with a lifetime mental disorder, only 15.7% had ever sought any form of help, with help-seeking rates for healthcare and non-healthcare settings being 7.6 and 8.1%, respectively. This observed help-seeking rate during their entire lifetime before the interview is higher than that in previous Chinese surveys in Xi’an city (4.7%) [21] and Shenzhen City (11.3%) [7]. However, the rate is much lower than that found in Western countries (range: 31.4% in Italy to 57.9% in the Netherlands) [22] and in people of Asian ancestry in the United States (25%) [23]. The current results showed that the proportion of help seekers in healthcare settings (7.6%) was roughly similar to the 8% found in the previous four provinces study in China [8] and the 6.7% found in the Shenzhen City survey [7]. The differences in overall help-seeking rates across Chinese surveys could be explained by different factors. First, the lower rate in Xi’an may be associated with its lower economic development and fewer mental health resources compared to Tianjin and Shenzhen [21]. Second, we included psychotic disorders in our survey, which are more likely to require some form of help/treatment than many other mental disorders, whereas the Xi’an and Shenzhen surveys did not include these disorders, which could have contributed to the lower help-seeking percentages in the latter [7, 21]. Apart from socio-economic factors, the described differences between the current results (and other findings from Chinese surveys) and those from surveys in western countries could be explained by higher levels of stigma toward mental disorders and lower mental health knowledge in China [24].

Relatives and colleagues/friends/neighbors were the most commonly reported source of help and the first choice to seek help for patients with mental disorders, which is consistent with previous work [25]. It has been shown that friends or relatives play an important role in helping patients deal with illness. In addition, they can help by recommending patients with a mental disorder to eventually seek professional help [26] or traditional, complementary and/or alternative medicine approaches [11]. Folk sources such as Qigong practitioners, witchdoctors and temples have traditionally been important providers of care for people with mental disorders in China and are still consulted widely. These practices are based on folk explanatory models that ascribe mental illnesses to an imbalance in the psychosocial, physiological and/or supernatural environment [11]. A previous study among Asian Americans found that 35% of patients with a lifetime mental disorder had visited religious/spiritual advisors [27]. However, the present study showed that only 0.7% of individuals with mental disorder sought help from such sources, and that in patients who did seek any help, no more than 0.9% first went to those sources. Interestingly, these rates are much lower than the rates observed for healthcare use and in the study among Asian Americans. It may be that seeking help from traditional sources has decreased over time or is underreported because respondents are reluctant to tell this to an interviewer with a medical/healthcare-related background.

Interestingly, of the patients who sought any healthcare services, a sizable proportion only sought non-mental healthcare services and 27.5% sought their first help in non-mental healthcare. Of the non-mental healthcare services, general hospitals and TCM hospitals were found to be the most common healthcare providers. The finding that TCM plays a significant role aligns with previous work. Two studies that were conducted in Taiwan showed that 9% of patients with schizophrenia and 40% of individuals with depression had used TCM services [28, 29]. In addition, the finding that many patients visit a general hospital aligns with previous work showing that patients with a mental disorder often visit general hospitals before they go on to visit mental health professionals [26]. Only a very small percentage of patients with a mental disorder in the current survey visited a community health center, although a previous study in Chengdu showed that 71.8% individuals in an urbanized community used services from a community health center during the past year [30]. The currently observed low usage rates might be explained by low awareness [31] and/or distrust in the quality of the provided service [32], and provides an indication of where possible improvements could be made.

We found that in the individuals with mental disorders, females are more likely to seek any form of help than males, but there were no sex differences in seeking help from either healthcare or non-healthcare sources. Differences in socialization of men and women could partly explain the differences in help-seeking, because women might be more likely to seek social support in response to stressful experiences than men [33]. When comparing age-groups, the present study found that older age-groups had lower odds to seek any help, but when they did, were more likely to seek help in healthcare than in non-healthcare settings. This result aligns with a previous study that has found that older generations are less likely to seek help for their mental disorders [34]. Marital status was presently found not to be related to any help-seeking behavior, but of persons who had ever sought any help, married individuals with mental disorder were more likely to seek help in healthcare than in non-healthcare settings compared to single, divorced or widowed patients. These results are in contrast to previous studies showing separated, widowed or divorced people with mental disorders to more often seek treatment than married individuals [1, 4]. However, the current findings are in line with the results of the survey in Xi’an [21]. The observed influence of marital status could be explained by a supportive role of a spouse that motivates a patient to seek treatment. Indeed, a previous study found that medical service use was increased by about 40% in the presence of a higher than median level of spousal support [35]. Per capita family income was presently observed to be related to help-seeking behavior, with lower income being associated with higher odds of seeking any help. However, income was unrelated to seeking help in healthcare vs. non-healthcare settings. This result is not consistent with findings from previous work in China that showed individuals with a lower income to have lower odds of help-seeking [3]. One explanation for this finding could be that low-income individuals may be more impaired by mental illness in their daily functioning than individuals with a higher income. An additional explanation for this finding could be that people with low income might be more likely to report mental-illness problems and help-seeking behavior than people with a high income.

Several clinical characteristics (psychotic disorder, organic mental disorders, having more than 1 mental disorder) were related with higher odds of help-seeking. This could be explained by the fact that these characteristics are indicative of considerable severity and severity is a known determinant of help-seeking [3, 5, 8]. Indeed, a previous study found that 90% of people with dementia in rural areas and 98% in urban areas sought treatment, and that 77% of individuals with schizophrenia in urban areas and 70% in rural areas had contact with mental health providers [36].

The current study found no clear association between perceived stigma and help-seeking behavior in individuals with mental disorders. This does not align with previous findings on the role of stigma. For instance, a community-based study in the US found that 25% of people who perceived a need for help did not seek services partly because they concerned about what others might think [37] and a US-based clinical study showed that higher perceived stigma is related to lower treatment adherence and higher discontinuation [38]. However, the current results are in line with previous work that found no relation between perceived public stigma and mental health service use [39]. The lack of an association between stigma and help-seeking in the current study might be explained by the fact that only perceived public stigma (i.e. other peoples’ perceived stigmatizing ideas/thoughts/actions) and not personal stigma (e.g., respondents’ own stigmatizing ideas/thoughts/actions) was assessed, whereas previous work found only personal stigma to be associated with help-seeking for mental health [39]. The current results did show an association between help-seeking and higher MHK. Indeed, a previous study in China showed the importance of knowledge in the process of help-seeking: they found that nearly 80% of a community resident sample had the intention to seek psychological help if needed, but only 12% knew of any hospitals or clinics that provide such help [9].

Although the current study had several strengths, including the survey design, extensive diagnostics, comprehensive help-seeking measurements and the inclusion of both demographic and clinical determinants, some study limitations should be considered. First, help-seeking was self-reported and recall bias or social desirability may have affected the responses. Second, a significant group of potentially interesting service users were not captured in this study (including subthreshold/subclinical patients) because only service use of those who were screened positive for high risk and met the criteria for a DSM disorder was investigated. In addition, diagnostic assessment was limited to DSM-IV Axis I disorders. DSM-Axis II disorders were not included in the SCID, whereas such disorders would likely be associated with significant need for care and help-seeking. Third, the results of the current study apply specifically to the Tianjin region and we should be careful with generalizing the findings directly to other regions/countries. Still, the results could give an indication of the kind of help-seeking patterns and correlates that would be found in comparable regions that have undergone similar rapid socioeconomic changes. Finally, the MHKQ used in this study mainly assessed basic mental health knowledge but not mental health literacy, which refers to knowledge and beliefs about mental disorders which aid their recognition, management or prevention [40]. Mental health literacy has previously been found to be related to seeking treatment [41]. Future study should pay more attention to the relation between mental health literacy and help-seeking behaviors.

The findings of current study have significant implication for the improvement of help seeking behavior for people with mental disorders. The results showed that the initial suggestion to seek help came mostly from relatives and colleagues/friends/neighbors. This shows that the social network and support of a patient play an important role in providing help for individuals with mental disorder in the community. This indicates that the social network should be considered as an important component when building mental health system in China. In addition, for non-mental healthcare settings, more programs are needed to improve the detection of mental health problems in western and TCM hospitals and general hospitals, to make sure that patients will receive needed care or can be referred to the appropriate mental healthcare providers. Finally, the results show that men, older people, those with high family income and those with common mental disorders are less likely to seek help and could be target groups for educational programs to improve help-seeking. Such actions would align with our finding that help-seeking was associated with higher mental health knowledge.

## Conclusions

Current findings provide important insights into the pattern and the correlates of help-seeking behavior of people with mental disorder in Tianjin which has undergone rapid economic development. This study showed that a small percentage of persons with mental disorders seek help. Of the patients who sought any healthcare services, a sizable proportion only sought non-mental healthcare services. Variations in the types of help-seeking may be partially explained by demographic and clinical characteristics. In addition, knowledge of mental health played an importance role in the process of help-seeking.

## Supplementary information

**Additional file 1.** The specific disorders included in each category.

**Additional file 2.** The items of the Mental Health Knowledge Questionnaire.

## Data Availability

All the data supporting our findings have been presented in the manuscript; the datasets used and/or analyzed during the current study are available from the corresponding author on reasonable request.

## Abbreviations

DSM-IV: Diagnostic and Statistical manual– fourth edition
GAF: Global Assessment of Functioning
GHQ-12: 12-item General Health Questionnaire
MHK: Mental Health Knowledge
MHKQ: Mental Health Knowledge Questionnaire
NOS: Not Otherwise Specified
PDD: the Perceived Discrimination and Devaluation scale
SCID: the Structured Clinical Interview for DSM-IV axis I disorders
TCM: Traditional Chinese Medicine
