# Supplementary material for: Help-seeking behaviors among Chinese people with mental disorders: a cross-sectional study
Source: BMC Psychiatry. 2019 Nov 29;19:373. doi: 10.1186/s12888-019-2316-z (PMC6883558; doi:10.1186/s12888-019-2316-z)
Supplement: Supplementary file 1 — Additional file 1. The specific disorders included in each category. [file 12888_2019_2316_MOESM1_ESM.docx]

**Additional file 1.** The specific disorders included in each category

| **Categories** | **Specific disorders** |
| --- | --- |
| ***Mood disorders*** | Bipolar disorder |
|  | Major depressive disorder |
|  | Dysthymic disorder |
|  | Depressive disorders NOS |
| ***Anxiety disorders*** | Panic disorder |
|  | Agoraphobia without panic |
|  | Social phobia |
|  | Specific phobia |
|  | Obsessive-compulsive disorder |
|  | Post- traumatic stress disorder |
|  | Generalized anxiety disorder |
|  | Anxiety disorders NOS |
| ***Substance use disorders*** | Alcohol use disorder |
|  | Sedative/hypnotic drug use |
| ***Psychotic disorders*** | Schizophrenia |
|  | Other Psychotic disorders |
|  | schizophreniform disorder |
|  | delusional disorder, |
|  | brief psychotic disorder |
|  | psychotic disorder NOS |
| ***Organic mental disorders*** | Mental retardation |
|  | Dementia |
|  | Mental disorders due to GMC or substance |
| ***Other DSM-IV mental disorders*** | Somatoform disorders |
|  | Adjustment disorder |
|  | Compulsive control disorders |
|  | Other DSM-IV axis I disorder |

GMC=general medical condition; NOS=not otherwise specified.
