# Supplementary material for: Help-seeking behaviors among Chinese people with mental disorders: a cross-sectional study
Source: BMC Psychiatry. 2019 Nov 29;19:373. doi: 10.1186/s12888-019-2316-z (PMC6883558; doi:10.1186/s12888-019-2316-z)
Supplement: Supplementary file 2 — Additional file 2. The items of the Mental Health Knowledge Questionnaire. [file 12888_2019_2316_MOESM2_ESM.docx]

**Additional file 2.** The items of the Mental Health Knowledge Questionnaire

| **items** | **answer** | |
| --- | --- | --- |
| 1. Mental health is an integral part of health. | **yes** | **no** |
| 2. Mental illnesses result from something wrong in thought. | **yes** | **no** |
| 3. Most people may have a mental problem, but they may not notice the problem. | **yes** | **no** |
| 4. Mental illnesses are all caused by stresses | **yes** | **no** |
| 5. Mental health includes normal intelligence, stable mood, harmonious relationships, and Good ability to adapt and so on. | **yes** | **no** |
| 6. Most mental illnesses cannot be cured. | **yes** | **no** |
| 7. If you suspect that you have mental problems or mental illnesses, you should go to a psychiatrist or psychologist for help | **yes** | **no** |
| 8. Individuals in any age can have a mental problem. | **yes** | **no** |
| 9. Mental illnesses or psychological problems cannot be prevented. | **yes** | **no** |
| 10. Even though a person is diagnosed with a severe mental disorder, he/she should take medication for only a short period rather than long-term continuous. | **yes** | **no** |
| 11. Optimistic attitude towards life, good interpersonal relationship and healthy lifestyle are helpful for us to keep good mental health. | **yes** | **no** |
| 12. It is more possible for a person with a family history of mental disorders to develop mental disorders or mental problems. | **yes** | **no** |
| 13. The mental problems in adolescent do not influence their academic achievement. | **yes** | **no** |
| 14. It is less likely to have mental problems or disorders in middle-age or older adults. | **yes** | **no** |
| 15. Someone with bad characters is more prone to have mental problems. | **yes** | **no** |
| 16. High psychological stress or major life events could induce mental problems or disorders. | **yes** | **no** |
